# Supplementary material for: Critical Factors Affecting the Prevalence of Staphylococcus aureus and Staphylococcal Enterotoxins in Raw Milk Cheese in the Alpine Region of Austria, Italy, and Switzerland
Source: Foods. 2025 Jun 21;14(13):2176. doi: 10.3390/foods14132176 (PMC12248861; doi:10.3390/foods14132176)
Supplement: Supplementary file 1 [file foods-14-02176-s001.zip › foods-3681234-supplementary.pdf]

Supplement Information

Table S1: Description of the cheese varieties from the Alpine regions of Austria (AT), Italy (IT), and Switzerland (CH) considered in this study. FDM = fat in dry matter, AOP/POD (Product of Designated Origin).

Variety	Description	Maximum water content [%]	FDM [%]	Temperature curd (scalding/holding) [°C]	pH (2 hours after molding)	Reference
**Österreichischer Alp- und Bergkäse (AT)**	hard cow's milk cheese, smear-ripened, up to 50 kg<br>regular distributed, sparse, round, pea-sized eyes<br>color: ivory to light yellow<br>origin: Vorarlberger Bergkäse	40	min. 45	min. 48	<6.2[1]	Codex Alimentarius Austriacus
**Schnittkäse (AT)**	semi-hard cow's milk cheese, smear-ripened, up to 4,5 kg<br>regular distributed, sparse, round lentil- to pea-sized or numerous split eyes<br>color: straw-yellow	46	45 (reference fat content)	approx. 36 to 39	<6.0[1]	Codex Alimentarius Austriacus
**Tilsiter (AT)**	semi-hard cow's milk cheese, smear-ripened, of approx. 3,5 kg<br>regular distributed, numerous, split eyes<br>color: straw-yellow	45	45 (reference fat content)	approx. 36 to 39	<6.0[1]	Codex Alimentarius Austriacus
**Alpkäse halbhart (CH)**	semi-hard cow's milk cheese, smear-ripened, between 5 and 16 kg<br>regular distributed, sparse, round rice- to maize-sized eyes<br>color: whitish ivory to yellowish<br>origin: Glarner Alpkäse AOP, Urner-, Schwyzer-, Bündner- und Nidwaldneralpkäse	38 to 41	45 to 54.9	min. 44, max. 47	<6.0	PDO specifications (www.aop-igp.ch/glarner-alpkaese-aop; www.schweizeralpkaese.ch/alpkaese-sorten)

1 [48]

**Vacherin Fribourgeois alpage AOP (CH)**	semi-hard cow's milk cheese, smear-ripened, between 6 and 10 kg<br>mostly irregular distributed, small eyes<br>color: ivory to light yellow<br>origin: canton of Fribourg	max. 46.5	48 to 54.9	30 to 36	<6.4[2]	PDO specifications (www.aop-igp.ch/vacherin-fribourgeois-aop)
**Formaggio d'alpe ticinese DOP (CH)**	semi-hard cow's milk cheese (addition of up to 30% goat's milk is possible), spontaneous colonization by filamentous fungi and yeasts, between 3 and 10 kg<br>regular distributed, sparse, round eyes, size not defined<br>color: light yellow to straw-yellow<br>origin: canton of Ticino	35 to 41	min. 45	41 to 50	<6.0[2]	PDO specifications (www.aop-igp.ch/formaggio-dalpe-ticinese-aop)
**Raclette d'alpage (CH)**	semi-hard cow's milk cheese, smear-ripened, between 4.3 and 5.7 kg, diameter of 29 to 32 cm<br>regular distributed, sparse, round, pea-sized eyes<br>color: not defined	40 to 44	50 to 54.9	37 to 42	<6.0[2]	https://www.schweizeralpkaese.ch/alp-erleben/regionen/region-wallis/
**Büscion, Büscion di Capra and Tomino di Capra (CH)**	fresh cow's or goat's milk cheese (semi-hard also possible), size not defined<br>color: whitish to pale yellow<br>origin: canton of Ticino	63	not available	24 to 26	pH 6.4 (pH final 4.4)	UCA advisory service, personal communication (https://www4.ti.ch/dfe/de/sa/consulenza-e-servizi/produzione-lattiero-casearia/), data sheet for Büscion is available
**Mutschli and Tomme du Valais (CH)**	semi-hard cow's milk cheese (ewe's or goat's milk is also possible), smear-ripened, between 0.5 and 4 kg<br>regular distributed, sparse to numerous, irregular round or split eyes<br>color: ivory to light yellow	40 to 44	50 to 54.9	40	<6.0[2]	www.patrimoineculinaire.ch/Produkte?text=mutschli&canton=&categorie=#435

[2] Agroscope advisory service, personal communication

**Formagella (CH)**	semi-hard cow's or goat's milk cheese, spontaneous colonization by grayish filamentous fungi sometimes with reddish-yellow coloration, size not defined<br>eyes not defined<br>color: whitish to straw-yellow	40 to 44	50 to 54.9	35 to 38	<6.2	UCA advisory service, personal communication (https://www4.ti.ch/dfe/de/sa/consulenza-e-servizi/produzione-lattiero-casearia/), data sheet available
**Minadur (IT)**	semi-hard cow's milk cheese, ripened at least 3 months, 5 to 7 kg<br>mostly irregular distributed small eyes<br>color: ivory to light yellow<br>origin: Val Brembana, Bergamo	45%	> 46%	43 to 46	not available	[83]<br>Picture:<br>https://shop.paganiantichisapori.it/formaggi-bergamaschi-di-latte-vaccino/482-ol-minadur.html
**Formagella della Valcamonica (IT)**	small cylindrical semi-hard cow's milk cheese (the dialect name "fomagéla" means "small cheese"), 18 to 22 cm in diameter and 4 cm in high, 1 to 1.5 kg<br>sparse round small eyes<br>color: white<br>origin: Val Camonica, Brescia	50%	> 43%	38 to 43	not available	PAT Lombardy Region<br>https://www.onaf.it/index.php?c=index&a=schedaformaggio&id=991
**Toma (IT)**	semi-hard cow's milk cheese of approx. 4 kg<br>sparse round, regularly distributed pea-sized eyes<br>origin: Verbania, Cusio, Ossola	45%	> 45%	41 to 44	not available	[84]<br>Picture:<br>https://prodottitipicivco.blogspot.com/2016/10/toma-del-mottarone.html

Table S2a: Samples: general information and raw milk treatment (sorted by "culture: direct or bulk," Table 2b, and "No."). Cheese types: FC = fresh cheese, SHC = semi-hard cheese, HC = hard cheese. Countries: AT = Austria, CH = Switzerland, IT = Italy, AOP/POD (Product of Designated Origin). Samples 13 and 135 (bold) were staphylococcal enterotoxin (SE)-positive. Samples 31, 90, and 92 (bold italic) were excluded from the statistical evaluation because of their high heat load.

General Information														Raw milk type							
sample Nr.	Cheese Type	Name of the product	Country											Animal			Storage		Treatment		
No	FC, SHC, HC	variety name	AT, CH, IT	region name	region group	AT1	AT2	CH1	CH2	CH3	IT1	IT2	IT3	Cow	Goat	Sheep	Storage [h]	Temperature [°C]	Yes/No [1]	Temperature [°C]	Time [sec]
34	SHC	Alpkäse halbhart	CH	Nidwalden	CH1			CH1						1			24	12	No	37	-
38	SHC	Alpkäse halbhart	CH	Glarus	CH2				CH2					1			12	11.2	No	37	-
39	SHC	Alpkäse halbhart	CH	Glarus	CH2				CH2					1			12	12.2	No	37	-
40	SHC	Alpkäse halbhart	CH	Graubünden	CH2				CH2					1			12	10	No	37	-
41	SHC	Alpkäse halbhart	CH	Schwyz	CH2				CH2					1			12	13	No	37	-
42	SHC	Alpkäse halbhart	CH	Schwyz	CH2				CH2					1			12	14	No	37	-
43	SHC	Alpkäse halbhart	CH	Glarus	CH2				CH2					1			12	12	No	37	-
44	SHC	Alpkäse halbhart	CH	Glarus	CH2				CH2					1			12	8.3	No	37	-
50	SHC	Alpkäse halbhart	CH	Glarus	CH2				CH2					1			10	12	No	37	-
51	SHC	Alpkäse halbhart	CH	Glarus	CH2				CH2					1			10	12	No	37	-
52	SHC	Alpkäse halbhart	CH	Glarus	CH2				CH2					1			10	12	No	37	-
56	SHC	Formaggio d'alpe ticinese AOP	CH	Ticino	CH3					CH3				1			12	8.6	No	37	-
62	SHC	Formaggio d'alpe ticinese AOP	CH	Ticino	CH3					CH3				1			12	9	No	37	-
72	SHC	Formaggio d'alpe ticinese AOP	CH	Ticino	CH3					CH3				1			12	9.5	No	37	-
73	SHC	Formaggio d'alpe ticinese AOP	CH	Ticino	CH3					CH3				1			12	10	No	37	-
74	SHC	Alpkäse halbhart	CH	Ticino	CH3					CH3					1				No	37	-
75	SHC	Formaggio d'alpe ticinese AOP	CH	Ticino	CH3					CH3				1	1		12	10	No	37	-
93	SHC	Alpkäse halbhart	CH	Graubünden	CH2				CH2					1			24	10	No	37	-
104	HC	Bergkäse	AT	Vorarlberg	AT2		AT2							1			12	11	No	-	-

105	HC	Bergkäse	AT	Vorarlberg	AT2		AT2							1			12	8	No	-	-
106	HC	Bergkäse	AT	Vorarlberg	AT2		AT2							1			12	12	No	-	-
107	HC	Bergkäse	AT	Vorarlberg	AT2		AT2							1			12	12	No	-	-
108	HC	Bergkäse	AT	Vorarlberg	AT2		AT2							1			10	11	No	-	-
109	HC	Bergkäse	AT	Vorarlberg	AT2		AT2							1			12	14	No	-	-
110	HC	Bergkäse	AT	Vorarlberg	AT2		AT2							1			9	10	No	-	-
111	HC	Bergkäse	AT	Vorarlberg	AT2		AT2							1			12	10.3	No	-	-
112	HC	Bergkäse	AT	Vorarlberg	AT2		AT2							1			7	13	No	-	-
113	HC	Bergkäse	AT	Vorarlberg	AT2		AT2							1			8	15	No	-	-
114	HC	Bergkäse	AT	Vorarlberg	AT2		AT2							1			12	11	No	-	-
115	HC	Bergkäse	AT	Vorarlberg	AT2		AT2							1			12	20	No	-	-
116	HC	Bergkäse	AT	Vorarlberg	AT2		AT2							1			12	9	No	-	-
117	HC	Bergkäse	AT	Vorarlberg	AT2		AT2							1			12	12	No	-	-
118	HC	Bergkäse	AT	Vorarlberg	AT2		AT2							1			10	12	No	-	-
119	HC	Bergkäse	AT	Vorarlberg	AT2		AT2							1			12	18	No	-	-
120	HC	Bergkäse	AT	Vorarlberg	AT2		AT2							1			12	14	No	-	-
121	HC	Bergkäse	AT	Vorarlberg	AT2		AT2							1			12	20	No	-	-
122	HC	Bergkäse	AT	Vorarlberg	AT2		AT2							1			12	16	No	-	-
124	SHC	Schnittkäse	AT	Tirol	AT1	AT1								1			6	6	No	-	-
125	SHC	Schnittkäse	AT	Tirol	AT1	AT1								1			8	7	No	-	-
127	SHC	Schnittkäse	AT	Tirol	AT1	AT1								1			12	8	No	-	-
128	SHC	Schnittkäse	AT	Tirol	ATT1	AT1								1			7	8	No	-	-
131	SHC	Schnittkäse	AT	Tirol	AT1	AT1								1			0		No	-	-
132	SHC	Schnittkäse	AT	Kärnten	AT1	AT1								1			0		No	-	-
**135**	**SHC**	**Tilsiter**	**AT**	**Tirol**	**AT1**	**AT1**								**1**			**0**		**No**	**-**	**-**
35	SHC	Alpkäse halbhart	CH	Obwalden	CH1			CH1						1			12	13	No	37	-
80	SHC	Formaggio d'alpe ticinese AOP	CH	Ticino	CH3					CH3				1	1		12	7.5	No	37	-
123	SHC	Schnittkäse	AT	Tirol	AT1	AT1								1			11	11	No	-	-

126	SHC	Schnittkäse	AT	Tirol	AT1	AT1								1			10		No	-	-
129	SHC	Schnittkäse	AT	Tirol	AT1	AT1								1			9.5	8	No	-	-
130	SHC	Schnittkäse	AT	Tirol	AT1	AT1								1			10	8	No	-	-
134	SHC	Schnittkäse	AT	Tirol	AT1	AT1								1			8.5	8	No	-	-
3	SHC	Minadur	IT	Val Brembana - Bergamo	IT1						IT1			1					No	37	-
5	SHC	Minadur	IT	Val Brembana - Bergamo	IT1						IT1			1					No	37	-
7	SHC	Minadur	IT	Val Brembana - Bergamo	IT1						IT1			1					No	37	-
8	SHC	Formagella	IT	Valcamonica - Brescia	IT2							IT2		1			12	18.5	No	37	-
10	SHC	Formagella	IT	Valcamonica - Brescia	IT2							IT2		1			12	14	No	37	-
11	SHC	Formagella	IT	Valcamonica - Brescia	IT2							IT2		1					No	37	-
**13**	**SHC**	**Formagella**	**IT**	**Valcamonica - Brescia**	**IT2**							**IT2**		**1**			**8**	**17**	**No**	**37**	**-**
14	SHC	Formagella	IT	Valcamonica - Brescia	IT2							IT2		1			12	12	No	37	-
16	SHC	Formagella	IT	Valcamonica - Brescia	IT2							IT2		1			12	12	No	37	-
18	SHC	Formagella	IT	Valcamonica - Brescia	IT2							IT2		1					No	37	-
20	SHC	Formagella	IT	Valcamonica - Brescia	IT2							IT2		1			12	15	No	37	-
22	SHC	Toma	IT	Verbania, Cusio, Ossola	IT3								IT3	1					No	42.5	600
23	SHC	Toma	IT	Verbania, Cusio, Ossola	IT3								IT3	1			24	6.8	No	37	-
25	SHC	Toma	IT	Verbania, Cusio, Ossola	IT3								IT3	1			24	6.8	No	37	-
26	SHC	Toma	IT	Verbania, Cusio, Ossola	IT3								IT3	1			24	6.8	No	37	-
27	SHC	Toma	IT	Verbania, Cusio, Ossola	IT3								IT3	1			24	6.8	No	37	-
28	SHC	Toma	IT	Verbania, Cusio, Ossola	IT3								IT3	1			24	6.8	No	37	-
29	SHC	Toma	IT	Verbania, Cusio, Ossola	IT3								IT3	1			24	6.8	No	37	-
30	SHC	Toma	IT	Verbania, Cusio, Ossola	IT3								IT3	1			24	6.8	No	37	-
31	***SHC***	***Mutschli/Tomme du Valais***	***CH***	***Bern***	***CH1***			***CH1***						***1***			***2***	***37***	***yes***	***72***	***15***
32	SHC	Mutschli/Tomme du Valais	CH	Bern	CH1			CH1						1			0	37	No	37	-
33	SHC	Alpkäse halbhart	CH	Uri	CH2				CH2					1			12	13	No	37	-
36	SHC	Mutschli/Tomme du Valais	CH	Bern	CH1			CH1						1			12	15	yes	60	10
37	SHC	Mutschli/Tomme du Valais	CH	Uri	CH2				CH2					1			12	8	No	37	-

45	SHC	Alpkäse halbhart	CH	Graubünden	CH2				CH2					1			24	10	No	37	-
46	SHC	Alpkäse halbhart	CH	Graubünden	CH2				CH2						1		8	8	yes	65	2
47	SHC	Alpkäse halbhart	CH	Graubünden	CH2				CH2						1		12	8.3	yes	65	1
48	SHC	Alpkäse halbhart	CH	Graubünden	CH2				CH2						1		12	8	yes	67	1
49	SHC	Mutschli/Tomme du Valais	CH	Graubünden	CH2				CH2						1		12	8	yes	65	1
53	SHC	Mutschli/Tomme du Valais	CH	Graubünden	CH2				CH2					1			12	12	No	37	-
54	SHC	Alpkäse halbhart	CH	Graubünden	CH2				CH2					1			12	12	No	37	-
55	SHC	Formagella	CH	Ticino	CH3					CH3					1		12	7	yes	61	1
57	SHC	Formagella	CH	Ticino	CH3					CH3				1			0	37	No	37	-
58	FC	Büscion, Büscion di Capra and Tomino di Capra	CH	Ticino	CH3					CH3					1		12		yes	61	1
59	SHC	Alpkäse halbhart	CH	Graubünden	CH2				CH2						1		10	8	No	37	-
60	FC	Büscion, Büscion di Capra and Tomino di Capra	CH	Ticino	CH3					CH3					1		10	8	No	37	-
61	SHC	Formagella	CH	Ticino	CH3					CH3					1		9	8	No	37	-
71	SHC	Mutschli/Tomme du Valais	CH	Bern	CH1			CH1						1			14	16	No	37	-
76	SHC	Mutschli/Tomme du Valais	CH	Bern	CH1			CH1						1			12	12	No	37	-
77	SHC	Formagella	CH	Ticino	CH3					CH3					1		12	7.5	No	37	-
78	FC	Büscion, Büscion di Capra and Tomino di Capra	CH	Ticino	CH3					CH3					1				No	37	-
79	FC	Büscion, Büscion di Capra and Tomino di Capra	CH	Ticino	CH3					CH3					1				No	37	-
81	SHC	Raclette d'alpage	CH	Valais	CH1			CH1						1			12	10	No	37	-
82	SHC	Raclette d'alpage	CH	Valais	CH1			CH1						1			12	12	No	37	-
83	SHC	Formagella	CH	Ticino	CH3					CH3					1		12	10	yes	62	60
84	SHC	Büscion, Büscion di Capra and Tomino di Capra	CH	Ticino	CH3					CH3					1		12	10	yes	62	60
85	SHC	Alpkäse halbhart	CH	Graubünden	CH2				CH2						1		10	8	No	37	-

86	SHC	Alpkäse halbhart	CH	Graubünden	CH2				CH2					1			24	9	No	37	-
87	SHC	Alpkäse halbhart	CH	Graubünden	CH2				CH2					1			24	10	No	37	-
88	SHC	Alpkäse halbhart	CH	Graubünden	CH2				CH2					1			12	10.5	No	37	-
89	SHC	Alpkäse halbhart	CH	Graubünden	CH2				CH2					1			12	9.5	No	37	-
90	***SHC***	***Alpkäse halbhart***	***CH***	***Ticino***	***CH3***					***CH3***					***1***		***72***	***4***	***yes***	***65***	***900***
92	***FC***	***Büscion, Büscion di Capra and Tomino di Capra***	***CH***	***Ticino***	***CH3***					***CH3***					***1***		***72***	***4***	***yes***	***65***	***900***
95	SHC	Vacherin Fribourgeois alpage AOP	CH	Fribourg	CH1			CH1						1			12	13	No	37	-
96	SHC	Vacherin Fribourgeois alpage AOP	CH	Fribourg	CH1			CH1						1			2	32	No	37	-
97	SHC	Vacherin Fribourgeois alpage AOP	CH	Fribourg	CH1			CH1						1			12	13	No	37	-
98	SHC	Raclette d'alpage	CH	Valais	CH1			CH1						1			12	7	No	37	-
103	SHC	Raclette d'alpage	CH	Valais	CH1			CH1						1			12	8	No	37	-
133	SHC	Schnittkäse	AT	Tirol	AT1	A1								1			5.5	7	No	-	-
1	SHC	Minadur	IT	Val Brembana - Bergamo	IT1						IT1			1					No	37	-
2	SHC	Minadur	IT	Val Brembana - Bergamo	IT1						IT1			1					No	37	-
4	SHC	Minadur	IT	Val Brembana - Bergamo	IT1						IT1			1					No	37	-
6	SHC	Minadur	IT	Val Brembana - Bergamo	IT1						IT1			1					No	37	-
9	SHC	Formagella	IT	Valcamonica - Brescia	IT2							IT2		1			12	14	No	37	-
12	SHC	Formagella	IT	Valcamonica - Brescia	IT2							IT2		1			8	17	No	37	-
15	SHC	Formagella	IT	Valcamonica - Brescia	IT2							IT2		1					No	37	-
17	SHC	Formagella	IT	Valcamonica - Brescia	IT2							IT2		1					No	37	-
19	SHC	Formagella	IT	Valcamonica - Brescia	IT2							IT2		1			12	15	No	37	-
21	SHC	Toma	IT	Verbania, Cusio, Ossola	IT3								IT3	1					No	37	-
24	SHC	Toma	IT	Verbania, Cusio, Ossola	IT3								IT3	1					No	37	-
63	SHC	Formaggio d'alpe ticinese AOP	CH	Ticino	CH3					CH3				1					No	37	-
99	FC	Büscion, Büscion di Capra and Tomino di Capra	CH	Ticino	CH3					CH3					1				No	37	-

100	FC	Büscion, Büscion di Capra and Tomino di Capra	CH	Ticino	CH3					CH3					1				No	37	-
101	SHC	Formagella	CH	Ticino	CH3					CH3					1				No	37	-
102	SHC	Formaggio d'alpe ticinese AOP	CH	Ticino	CH3					CH3				1					No	37	-

Notes:

Table elements were left blank if no data were available or it made no sense to fill them in. [1)] “No” means raw milk.

Table S2b: Samples: culture types and cheese production parameters (sorted by "culture: direct or bulk," and "no."). Samples 13 and 135 (bold) were staphylococcal enterotoxin (SE)-positive. Samples 31, 90, and 92 (bold italic) were excluded from the statistical evaluation because of their high heat load.

General Information	Cheesemaking																															
Sample No.	Added culture						Pre-maturation		Coagulation		Cutting		Water addition			Maximum applied temperature (scalding and others)			Holding			Pressing					Ripening planned	sum of all temp-time 7.48°C 2)		graph: sum of all temp-time >48 °C 2)		Graph 3)
No.	culture: original type	culture: corrected brand name	culture: meso/thermophilic	culture: direct or bulk starter	origin: no/commercial/natural	adding point 2)	Temperature [°C]	Time [min]	Temperature [°C]	Time [min]	Temperature [°C]	Time [min]	Yes/No	%	Temperature [°C]	Yes/no	Temperature [°C]	Time [min]	Yes/no	Temperature [°C]	Time [min]	Yes/no	Temperature [°C]	Time [min]	pH after 2 h	pH before salting	Weeks	max Temperature [°C]	Time [log s]	max Temperature [°C]	Time [log s]	Temp-time (ϑ*t) [°C*s]
34	Fidenza, RMK291	Liebefeld Kulturen RMK291/Labeco Fidenza	T	bulk	commercial	morning	31	30	31	30	31	25	No	-	-	Yes	42	30	Yes	42	5	yes	-	420	5.15	5.15	14	42	5.074816441			213.1
38	Glarner Alpkäsekultur	Glarner Alpkäsekultur (basis Liebefeld Kulturen 291/yog/401)	MT	bulk	commercial	morning	32.1	20	32.1	35	32.1	12	No	-	-	Yes	45.5	30	Yes	45.5	25	yes	-	480	-	5.17	8	45.5	4.899382706			222.9
39	Glarner Alpkäsekultur	Glarner Alpkäsekultur (basis Liebefeld Kulturen 291/yog/401)	MT	bulk	commercial	morning	32	25	32	30	32	20	No	-	-	Yes	45.5	35	Yes	45.5	25	yes	-	480	-		8	45.5	4.903632516			223.1
40	401, RMK291	Liebefeld Kulturen RMK291/Liebefeld Kulturen MK401	MT	bulk	commercial	morning	25	30	25	35	25	20	yes	10	40	Yes	42	25	Yes	42	20	yes	-	480		5.2	6	42	4.902002891			205.9
41	RMK Labeco	Urner Alpkultur (basis Liebefeld Kulturen 291/yog/401)	MT	bulk	commercial	morning	32	30	32	33	32	30	yes	7	60	Yes	45	35	Yes	45	15	yes	-	15	5.73	5.18	10	45	4.721645766			212.5
42	FSK	Natural whey starter	T	bulk	natural	morning	32.6	30	32.6	33	32.6	30	yes	5	10	Yes	46	45	Yes	46	15	yes	-	135	-		10	46	4.781611782			220.0
43		Glarner Alpkäsekultur (basis Liebefeld Kulturen 291/yog/401)	MT	bulk	commercial	morning	32	20	32	30	32	30	No	-	-	Yes	45.1	45	Yes	45.1	20	yes	-	420	6.3		8	45.1	4.887054378			220.4
44	Glarner Alpkäsekultur	Glarner Alpkäsekultur (basis Liebefeld Kulturen 291/yog/401)	MT	bulk	commercial	morning	26.5	39	31.9	30	31.9	34	No	-	-	Yes	44.8	40	Yes	44.6	13	yes	-	5	5.87	5.2	8	44.8	4.723127159			211.6
50	Glarner Alpkäsekultur	Glarner Alpkäsekultur (basis Liebefeld Kulturen 291/yog/401)	MT	bulk	commercial	morning	-	30	-	32	-	25	No	-	-	Yes	45	40	Yes	45	20	yes	-	480	-		8	45	4.866995813			219.0
51	Glarner Alpkäsekultur	Glarner Alpkäsekultur (basis Liebefeld Kulturen 291/yog/401)	MT	bulk	commercial	morning	-	30	-	32	-	25	No	-	-	Yes	45	40	Yes	45	20	yes	-	480	-		8	45	4.866995813			219.0

52	Glarner Alpkäsekultur	Glarner Alpkäsekultur (basis Liebefeld Kulturen 291/yog/401)	MT	bulk	commercial	morning	-	30	-	32	-	25	No	-	-	Yes	45	40	Yes	45	20	yes	-	480	-		8	45	4.866995813			219.0
56	Vegetativa Dop	UCA Vegetativa (basis Liebefeld Kulturen 291/3008/401)	MT	bulk	commercial	morning	28	60	32.5	35	32.5	15	yes	10	32.5	Yes	45	40	Yes	45	15	yes	-	1200	-	5.17	8	45	5.09725731			229.4
62	Vegetativa Dop	UCA Vegetativa (basis Liebefeld Kulturen 291/3008/401)	MT	bulk	commercial	morning	33	40	33	30	33	10	yes	9	15	Yes	46	40	Yes	46	10	no	-		-		-	46	4.707570176			216.5
72	UCA RM-Kultur	UCA Vegetativa (basis Liebefeld Kulturen 291/3008/401)	MT	bulk	commercial	morning	2.5	35	32.5	35	32.5	18	No	-	-	yes	46	40	yes	46	18	yes	-	1200	-	5.35	8	46	5.093281568			234.3
73	UCA Vegetativa	UCA Vegetativa (basis Liebefeld Kulturen 291/3008/401)	MT	bulk	commercial	morning	33	85	33	38	33	12	yes	15	41	yes	47	60	yes	47	18	yes	-		-	5.3	8	46	4.748032894			218.4
74	UCA RM-Kultur	UCA Vegetativa (basis Liebefeld Kulturen 291/3008/401)	MT	bulk	commercial	morning	35	30	35	30	35	10	No	-	-	yes	43	20	yes	43	30	yes	-	600	-	5.3	8	43	4.635483747			199.3
75	UCA RM-Kultur	UCA Vegetativa (basis Liebefeld Kulturen 291/3008/401)	MT	bulk	commercial	morning	32	40	32	32	32	12	yes	8	41	yes	45	36	yes	45	8	no	-		-	5.3	-	45	4.706547103			211.8
93	302, 401	Liebefeld Kulturen RMK302/Liebefeld Kulturen MK401	MT	bulk	commercial	morning		40	-	40	-	20	yes	-	20	yes	42.5	30	yes	42.5	30	-	-		-		-	42.5	4.982271233			211.7
104	Bulk starters, S2 (thermophil), MKR (thermophil), Casei 01 (mesophil)	S2 (HBLFA Tirol), MKR (HBLFA Tirol), LC-01 (Chr Hansen)	MT	bulk	commercial	morning	25	50	32	30	32	20	Yes	2	48	Yes	52	50	Yes	51	5	yes	30	1080	-		52 to 104			52	1.740362689	90.5
105	Bulk starters, S2 (thermophil), MKR (thermophil), Casei 01 (mesophil)	S2 (HBLFA Tirol), MKR (HBLFA Tirol), LC-01 (Chr Hansen)	MT	bulk	commercial	morning	30	40	31.8	45	31.8	10	Yes	8	48	Yes	52	40	Yes	52	4	yes	30	1200	-		24			52	1.643452676	85.5
106	Bulk starters, S2 (thermophil), MKR (thermophil), Casei 01 (mesophil)	S2 (HBLFA Tirol), MKR (HBLFA Tirol), LC-01 (Chr Hansen)	MT	bulk	commercial	morning	25	60	32	40	32	10	Yes	2.5	48	Yes	52	50	Yes	52	15	yes	30	1080	-		24			52	1.812913357	94.3
107	Bulk starters, S2 (thermophil), MKR (thermophil), Casei 01 (mesophil)	S2 (HBLFA Tirol), MKR (HBLFA Tirol), LC-01 (Chr Hansen)	MT	bulk	commercial	morning	25	60	32	40	32	10	Yes	10	48	Yes	52.5	65	Yes	52.5	5	yes	30	1080	-		24			52.5	1.84509804	96.9
108	Bulk starters, S2 (thermophil), MKR (thermophil), Casei 01 (mesophil)	S2 (HBLFA Tirol), MKR (HBLFA Tirol), LC-01 (Chr Hansen)	MT	bulk	commercial	morning	11 to 32	57	32	40	32	10	Yes	5	48	Yes	52	50	Yes	52	10	yes	30	1200	-		24			52	1.77815125	92.5
109	Bulk starters, S2 (thermophil), MKR (thermophil), Casei 01 (mesophil)	S2 (HBLFA Tirol), MKR (HBLFA Tirol), LC-01 (Chr Hansen)	MT	bulk	commercial	morning	14 to 32	45	32	40	32	22.5	Yes	10	48	Yes	52	45	Yes	52	10	yes	30	1200	-		24 to 52			52	1.740362689	90.5
110	Bulk starters, S2 (thermophil), MKR (thermophil), Casei 01 (mesophil)	S2 (HBLFA Tirol), MKR (HBLFA Tirol), LC-01 (Chr Hansen)	MT	bulk	commercial	morning	10 to 32	60	31	40	31.8	10	No	-	-	Yes	53	45	Yes	52	10	yes	27.5	1080	-		24			53	1.740362689	92.2
111	Bulk starters, S2 (thermophil), MKR (thermophil), Casei 01 (mesophil)	S2 (HBLFA Tirol), MKR (HBLFA Tirol), LC-01 (Chr Hansen)	MT	bulk	commercial	morning	10 to 32	60	32	35	32	15	Yes	5	53	Yes	53	45	Yes	51.5	10	yes	30	1140	-		24			53	1.740362689	92.2
112	Bulk starters, S2 (thermophil), MKR (thermophil), Casei 01 (mesophil)	S2 (HBLFA Tirol), MKR (HBLFA Tirol), LC-01 (Chr Hansen)	MT	bulk	commercial	morning	13 to 31	30	31	40	31	10	Yes	5	48	Yes	52	45	Yes	52	10	yes	27.5	1140	-		24			52	1.740362689	90.5
113	Bulk starters, S2 (thermophil), MKR (thermophil), Casei 01 (mesophil)	S2 (HBLFA Tirol), MKR (HBLFA Tirol), LC-01 (Chr Hansen)	MT	bulk	commercial	morning	25	40	32	40	32	10	Yes	6	47	Yes	52	45	Yes	52	10	yes	30	1020	-		24			52	1.740362689	90.5
114	Bulk starters, S2 (thermophil), MKR (thermophil), Casei 01 Lochbildungskultur (mesophil), Sennereikultur	S2 (HBLFA Tirol), MKR (HBLFA Tirol), LC-01 (Chr Hansen)	MT	bulk	commercial	morning	25 to 31	40	31	40	31	-	Yes	8	49	Yes	52	60	Yes	52	1	yes	27.5	1200	-		min. 12			52	1.785329835	92.8

115	"wild" culture (meso- und thermophil), "wild" lab	-	MT	bulk	natural	morning/evening	25 to 30	25	31.2	45	31.2	15	Yes	2	42	Yes	53	50	No	-		yes	27.5	1200	-		min. 12			53	1.698970004	90.0
116	Bulk starters, S2 (thermophil), MKR (thermophil), Sennereikultur	S2 (HBLFA Tirol), MKR (HBLFA Tirol)	MT	bulk	commercial	morning	18 to 32	40	32	40	32	3	No	-	-	Yes	53	55	Yes	51.5	5	yes	27.5	1200	-		min. 12			53	1.77815125	94.2
117	Bulk starters, S2 (thermophil), MKR (thermophil)	S2 (HBLFA Tirol), MKR (HBLFA Tirol)	MT	bulk	commercial	morning	25 to 30	30	31	35	31	15	Yes	2.5	47	Yes	52	45	Yes	51	5	yes	27.5	1200	-		min. 12			52	1.698970004	88.3
118	Bulk starters, S2/01 Casei (thermophil), MKR (thermophil)	S2 (HBLFA Tirol), MKR (HBLFA Tirol), LC-01 (Chr Hansen)	T	bulk	commercial	morning	33	55	32	32	32	5	No	-	-	Yes	52	60	Yes	52	5	yes	37.5	1200	-		min. 12			52	1.812913357	94.3
119	Bulk starter, "wild" culture (thermophil), "wild" lab	-	T	bulk	natural	morning/evening	32	45	32	40	32	10	Yes	3	48	Yes	52	45	Yes	52	10	yes	32.5	1200	-		min. 12			52	1.740362689	90.5
120	2 Bulk starters (thermophil), Sennereikultur	-	T	bulk	-	morning	30	40	30	35	32	5	Yes	1	52.3	Yes	52.3	60	Yes	52	2	yes	27.5	1200	-		-			52.3	1.792391689	93.7
121	Bulk starter, "wild" culture (thermophil), "wild" lab	-	T	bulk	natural	morning/evening	25	45	32	38	32	25	Yes	10	48	Yes	52	45	Yes	52	5	yes	27.5	1200	-		12			52	1.698970004	88.3
122	Bulk starter, S2/01 Casei (thermophil), "wild" lab	S2 (HBLFA Tirol), LC-01 (Chr Hansen)	T	bulk	commercial	morning/evening	25	20	31.5	30	31.5	5	No	-	-	Yes	52	45	Yes	52	10	yes	27.5	1200	-		min. 12			52	1.740362689	90.5
124	Bulk starters, Flora danica (mesophil), CHIV-19 (mesophil)	Flora danica (Chr Hansen), CHIV-19 (Chr Hansen)	M	bulk	commercial	morning	30	60	32	45	32	20	Yes	10	35	No	38	20	Yes	38	20	yes	37	120	5.3		5 to 6	38	4.23299611			160.9
125	Bulk starters, CHN19/Flora danica (mesophil), Probat 222 (mesophil)	Flora danica (Chr Hansen), CHIV-19 (Chr Hansen), Probat 222 (Chr Hansen)	M	bulk	commercial	morning	20 to 32	50	32	45	31.8	20	Yes	10	35	No	38	10	Yes	38	10	yes	34.2	300	5.9		5	38	4.739572344			180.1
127	1 Bulk starter (mesophil)	-	M	bulk	-	morning	32	60	32	40	32	25	Yes	10	37	No	37	35	Yes	37	35	no	-		5.65		4	37	4.739572344			175.4
128	1 Bulk starter, Flora danica (mesophil)	Flora danica (Chr Hansen)	M	bulk	commercial	morning	32	90	32	25	32	15	Yes	30	45	No	36	20	Yes	36	20	yes	35	1080	5.7		6	36	5.000867722			180.0
131	2 Bulk starters, Flora danica (mesophil), CHN19 (mesophil)	Flora danica (Chr Hansen), CHN-19 (Chr Hansen)	M	bulk	commercial	morning	25	60	32	45	32	20	Yes	7.5	40	No	37	60	Yes	37	60	yes	-	30	5.3		5 to 6	37	4.217483944			156.0
132	2 Bulk starters, Flora danica (mesophil), MKR (thermophil; added at coagulation)	Flora danica (Chr Hansen), MKR (HBLFA Tirol)	MT	bulk	commercial	morning	33	45	32	40	31.5	15	Yes	35	35	No	39	20	Yes	39	20	yes	-	1260	<6		4 to 6	39	4.924279286			192.0
**135**	**1 Bulk starter, Flora danica (mesophil) - culture can be 3-4 days old**	**Flora danica (Chr Hansen)**	**M**	**bulk**	**commercial**	**morning**	**32**	**40**	**32**	**35**	**32**	**10**	**Yes**	**15**	**40**	**No**	**37**	**17.5**	**Yes**	**37**	**17.5**	**no**	**-**		**6.52**		**4**	**37**	**3.857332496**			**142.7**
35	RMK2020, Danesco Helvetica	Liebefeld Kulturen RMK2020/Danisco LH100	MT	bulk + direct	commercial	morning	31.8	45	31.8	37	31.8	35	yes	10	35	Yes	47.3	35	Yes	47.3	10	yes	-	240	5.38	5.20	9	47.3	4.828144107			228.4
80	Danisco Choozit, 4001	UCA Vegetativa (basis Liebefeld Kulturen 291/3008/401)/Danisco 4001	MT	bulk + direct	commercial	morning	33	20	33	30	33	10	No	-	-	yes	45.5	55	yes	55.5	10	yes	-	360	-	5.3	8			55	1.812913357	99.7
123	Bulk starter, CHN-19 (mesophil) and direct Starter Alp D (thermophil, no activation)	CHN-19 (Chr Hansen), Alp D (Danisco)	T	bulk + direct	commercial	morning/evening	32	40	32	35	32	30	Yes	1	30	No	37	30	Yes	37	30	no	-		5.3		6 to 7	37	4.694605199			173.7
126	Bulk starter CHN19 (mesophil) and direct starter Alp Dip (thermophil)	CHN-19 (Chr Hansen), Alp D (Danisco)	MT	bulk + direct	commercial	morning/evening	25	35	33	45	32	30	Yes	15	-	No	-	30	Yes	-	30	yes	-	600	5.8		-	33	4.914871818			162.2
129	Bulk starters, R 708 (mesophil), CHN19 (mesophil); and direct starters Lb. helv. + Lb. lactis subsp. lactis (thermophil) before coagulation	R708 (Chr Hansen), CHN-19 (Chr Hansen)	MT	bulk + direct	commercial	morning/evening	12 to 32	80	32	35	32	30	Yes	12.5	42	No	38	35	Yes	38	35	yes	33	240	6.02		5 to 6	38	4.788875116			182.0

130	Bulk starters, CHN19/Flora danica (mesophil), LH-B02+Lyofast MT092 (thermophil)	CHN-19 (Chr Hansen), Flora danica (Chr Hansen), LH-B02 (Chr Hansen), Lyofast MT092 (Clerici Sacco)	MT	bulk + direct	commercial	morning	22	60	32	37.5	32	40	Yes	7.5	50	No	36	35	Yes	36	35	yes	23	1200	5.95		6 to 7	36	5.080806804			182.9
134	1 Direct starter prior milking in the morning, AlpD (mesophil, thermophil) for 250 l to 700 l milk; 1 Bulk starter CHN19 or Flora danica (mesophil)	Alp D (Danisco), CHN-19 (Chr Hansen), Flora danica (Chr Hansen)	MT	bulk + direct	commercial	morning	18 to 32	35	32	35	31.6	20	Yes	17.5	37	No	37	45	Yes	37	45	no	-		5.8		5	37	4.617000341			170.8
3	Direct starter	Cr. Hansen CO-03 Thermophilic culture	T	direct	commercial	morning	33	45	36	30	36	10	No	-	-	Yes	44	30	No			yes	42	720			14	44	4.699837726			206.8
5	Direct starter	Cr. Hansen CO-02 Thermophilic culture	T	direct	commercial	morning	30	100	35	35	34	10	No	-	-	Yes	34	22	Yes	35.6	16	yes	40	1440			14	40	4.98846977			199.5
7	Direct starter	Cr. Hansen CO-03 Thermophilic culture	T	direct	commercial	morning	33	45	36	30	36	10	No	-	-	Yes	46	30	No			yes	42	720			14	46	4.699837726			216.2
8	Direct starter	Sacco ST031 Thermophilic culture	T	direct	commercial	morning	38	35	38	25	37	3	No	-	-	Yes	41.5	5	Yes	40	11	yes	24	270	5.45	5.39	4	41.5	4.807128956			199.5
10	Autochtonous starter	MIFISSO (autochtonous culture)		direct	commercial	morning	38	30	38	15	38	5	No	-	-	Yes	40	5	Yes	40	5	yes	40	420	5.50	5.10	4	40	4.857332496			194.3
11	Autochtonous starter	MIFISSO (autochtonous culture)		direct	commercial	morning	37	5	37	43	38	20	No	-	-	Yes	38	16	Yes	41	25	yes	38	270	5.39	5.34	4	41	4.35679046			178.6
**13**	**Autochtonous starter**	**MIFISSO (autochtonous culture)**		**direct**	**commercial**	**morning**	**37**	**20**	**39**	**55**	**38.5**		**No**	**-**	**-**	**Yes**	**42.6**	**10**	**Yes**	**42**	**10**	**yes**	**26**	**240**	**6.42**	**5.84**	**4**	**43.6**	**4.689308859**			**204.5**
14	Autochtonous starter	MIFISSO (autochtonous culture)		direct	commercial	morning	34		37	50	37	5	No	-	-	Yes	41	5	Yes	41	17	yes	33		6.15	5.34	4	41	4.679609572			191.9
16	Direct starter	PRODOR Soft cheese		direct	commercial	morning	34		37	50	37	5	No	-	-	Yes	41	5	Yes	41	17	yes	40		5.99	5.29	4	41	4.679609572			191.9
18	Autochtonous starter	MIFISSO (autochtonous culture)		direct	commercial	morning	38	40	39	20	39	25	No	-	-	Yes	43.3	5	Yes	40	20	yes	30	100	6.06	5.30	4	43.3	4.100370545			177.5
20	Autochtonous starter	MIFISSO (autochtonous culture)		direct	commercial	morning	40	30	40.6	15	37.5	5	No	-	-	Yes	40	5	Yes	40	6	yes	34	410	5.60	5.10	4	40	4.854063012			194.2
22	Direct starter	Thermophilic culture (St. thermophilus, Lb. helveticus)	T	direct	commercial	morning	42	40	42	20	42		No	-	-	No			Yes	38	10	yes		1200			4	42	4.881954971			205.0
23	Direct starter	Bioagro TB1/BD		direct		morning	39.5	40	38.5	35	38.5		No	-	-	Yes	43.5		Yes	38.5		yes	20	2400	5.40	5.50	24	43.5	5.171726454			225.0
25	Direct starter	BioAgro MW2		direct		morning	39.5	31	39.5	35	39.6		No	-	-	Yes	42.5		Yes	39.5		yes	18	2400	5.27	5.29	24	42.5	5.170144323			219.7
26	Direct starter	BioAgro MW2		direct		morning	39	33	39	35	38.5		No	-	-	Yes	41.6		Yes	39.2		yes	19	2400	6.40	5.55	24	41.6	5.170496406			215.1
27	Direct starter	Bioagro TB1/BD		direct		morning	39.5	33	38.5	35	38.5		No	-	-	Yes	43.5		Yes	38.6		yes	18.8	2400	6.54	5.67	24	43.5	5.170496406			224.9
28	Direct starter	Bioagro TB1/BD		direct		morning	39.5	40	38.5	35	38.5		No	-	-	Yes	42.5		Yes	39		yes	19.2	2400	5.37	5.30	24	42.5	5.171726454			219.8
29	Direct starter	Thermophilic culture	T	direct	commercial	morning	39.5	40	38.5	35	38.5		No	-	-	Yes	43.5		Yes	38.5		yes	20	2400	5.40	5.40	4	43.5	5.171726454			225.0
30	Direct starter	Bioagro TB1/BD		direct		morning	39.5	40	38.5	35	38.5		No	-	-	Yes	43.5		Yes	38.5		yes	20	2400	5.40	5.30	24	43.5	5.171726454			225.0
31	***4001***	***Danisco 4001***	***MT***	***direct***	***commercial***	***morning***	***32***	***30***	***32***	***34***	***32***	***20***	***Yes***	***30***	***53***	***Yes***	***38***	***15***	***Yes***	***38***	***12***	***yes***	***-***	***3***	***-***		***3***			***72***	***1.176091259***	***84.7***
32	4002	Danisco 4002	MT	direct	commercial	morning	33	40	33	40	33	25	Yes	30	43	Yes	35	35	Yes		3	yes	-	180	-	5	3	35	4.287353773			150.1
33	4001	Danisco 4001	MT	direct	commercial	morning	32	50	32	35	32	30	No	-	-	Yes	42	35	Yes	42	15	yes	-	1800	-	5.12	6	42	5.20709554			218.7
36	4001	Danisco 4001	MT	direct	commercial	morning	30	30	30	30	30	20	yes	30	55	Yes	38	20	Yes	38	10	no	-	-	-		3			60	1	60.0
37	4001	Danisco 4001	MT	direct	commercial	morning	33	40	33	35	33	35	yes	7	-	Yes	44	35	Yes	44	20	yes	-	1320	-	5.4	3.5	44	5.121559844			225.3

45	MT092	Sacco MT092	MT	direct	commercial	morning	31	30	31	32	31	30	yes	10	40	Yes	45	22	Yes	45	22	yes	-	480	5.7	5.3	6	45	5.091174361			229.1
46	Alp Dip	Danisco alp D	MT	direct	commercial	morning	32	30	32	40	32	20	yes	7	40	Yes	39	15	Yes	39	5	no	-		-	5.17	3			65	0.301029996	19.6
47	Alp Dip, MT092	Danisco alp D/Sacco MT091	MT	direct	commercial	morning	33	60	35	32	35	20	No	-	-	Yes	39	20	Yes	39	5	no	-		-	5.2	3			65	0	0.0
48	MT092	Sacco MT092	MT	direct	commercial	morning	33	40	33	33	33	15	yes	10	-	Yes	-	46	Yes	-	5	no	-		-	5.2	3			67	0	0.0
49	MT092	Sacco MT092	MT	direct	commercial	morning	33	40	33	33	33	15	yes	15	60	Yes	36	15	Yes	36	5	no	-		-	5.2	3			65	0	0.0
53	Alp Dip	Danisco alp D	MT	direct	commercial	morning	30	30	30	35	30	30	yes	7	0.61	Yes	41	30	Yes	41	25	no	-		-		4	45	4.717670503			212.3
54	Alp Dip	Danisco alp D	MT	direct	commercial	morning	30	30	30	35	30	30	yes	7	41	Yes	41	30	Yes	41	25	no	-		-		6	41	4.717670503			193.4
55	Micromilk T1/T2 Direktstarter	Micromilk T1/T2	T	direct	commercial	morning	39	30	39	30	-	-	No	-	-	Yes	39	15	no	-	-	no	-		-		4			61	0	0.0
57	ST020	Sacco ST020	T	direct	commercial	morning	35	30	35	35	375	10	No	-	-	Yes	38	13	no	-		no	-		-		-	38	3.722633923			141.5
58	4001	Danisco 4001	MT	direct	commercial	morning	28	720	-	-	-	-	No	-	-	no	-	-	no	-		yes	-	720	-	4.5	-			61	0	0.0
59	Kazu1, 4001, Alp Dip	Danisco KASU/Danisco 4001/Danisco alp D	MT	direct	commercial	morning	-	30	-	40	-	20	yes	8	-	Yes	42	30	Yes	-		no	-		-	5.1	6	46	4.635483747			213.2
60	ME2 Micromilk	Micromilk ME2	M	direct	commercial	morning	20	5	23.8	60	20	1260	No	-	-	no	-		no	-		yes	-	1440	-	4.4	-	23.8	5.305136319			126.3
61	Lyo82	Sacco 082B	T	direct	commercial	morning	35	75	35	45	33	25	No	-	-	Yes	35		no	-		no	-		-		3	35	4.613841822			161.5
71	4001	Danisco 4001	MT	direct	commercial	morning	-	40	-	35	-	30	yes	33	55	yes	55	39	yes	55	7	yes	-	1200	-		3			55	1.662757832	91.5
76	5DCU	Danisco 4001	MT	direct	commercial	morning	32	40	32	30	32	25	yes	33	60	yes	60	40	yes	60	8	yes	-	240	-		3			60	1.681241237	100.9
77	Y082B	Sacco 082B	T	direct	commercial	morning	35	90	35	30	35	10	No	-	-	yes	35		yes	-	7.5	no	-		-	5.2	4	35	4.711385379			164.9
78	MW080, Geo13	Sacco MW030/Danisco Geo13	M	direct	commercial	morning	28	90	28	720	28	720	No	-	-	no	-		no	-		no	-		-	4.27	1	28	4.962842681			139.0
79	MOS 062B, Geo13	Sacco MOS062/Danisco Geo13	MT	direct	commercial	morning	28	90	28	720	28	720	No	-	-	yes	28	720	yes	28	1440	no	-		-	4.62	1	28	5.345177617			149.7
81	Alp Dip	Danisco alp D	MT	direct	commercial	morning	25	35	25	33	25	10	No	-	-	yes	42	30	yes	42	25	yes	-	1800	-	5.36	8	42	5.2018885			218.5
82	Mobact, Choozit	Danisco Probat	M	direct	commercial	morning	25	40	25	40	25	15	No	-	-	yes	43	26	yes	43	15	yes	-	1800	-		8	43	5.202379321			223.7
83	Y080, ST020	Sacco Yo80B/ST020	T	direct	commercial	morning	38	50	36.5	38	36.5	15	No	-	-	yes	38.5	6	yes	38.5	5	no	-		-	5.4	3			62	1.77815125	110.2
84	Choozit, 4001	Danisco 4001	MT	direct	commercial	morning	27	60	27	720	27	720	No	-	-	no	-		no	-	-	yes	-	2880	-	4.2	2			62	1.77815125	110.2
85	Kazu1, 4001, Alp Dip	Danisco KASU/Danisco 4001/Danisco alp D	MT	direct	commercial	morning	-		-		-		-	-	-	-	-		-	-		-	-		-		6	8	4.556302501			36.5
86	Alp Dip	Danisco alp D	MT	direct	commercial	morning	20	60	32	37	32	30	yes	7	34	yes	45	35	yes	45	16	no	-		5.9	5.2	24	45	4.987129768			224.4
87	Alp Dip	Danisco alp D	MT	direct	commercial	morning	32.3	60	35	36	35	27	yes	6	35	yes	45	30	yes	45	27	no	-		5.9	5.2	24	45	4.987666265			224.4
88	Alp Dip	Danisco alp D	MT	direct	commercial	morning	32	30	32	30	32	31	No	-	-	yes	44	30	yes	44	30	yes	-	18	6	5.3	5	44	4.727053011			208.0
89	Alp Dip	Danisco alp D	MT	direct	commercial	morning	32	30	32	30	32	30	yes	7	35.1	yes	44	30	yes	44	35	yes	-	15	6	5.3	5	44	4.727541257			208.0
90	***4001***	***Danisco 4001***	***MT***	***direct***	***commer-cial***	***morning***	***32***	***30***	***32***	***40***	***32***	***3***	***yes***	***10***	***34***	***yes***	***42***	***20***	***yes***	***42***	***30***	***yes***	***-***	***600***	***-***	***5.2***	***6***			***65***	***2.954242509***	***192.0***
92	***Mesofila Micromilk***	***Micromilk ME***	***M***	***direct***	***commer-cial***	***morning***	***-***	***-***	***-***	***-***	***-***	***1440***	***No***	***-***	***-***	***-***	***-***	***-***	***-***	***-***	***-***	***yes***	***-***	***2880***	***-***		***-***			***65***	***2.954242509***	***192.0***
95	Flora, Probat	Hansen Flora Danica/Danisco Probat	M	direct	commercial	morning	-	25	-	45	-	10	yes	8	52.5	yes	35	80	no	-		yes	-	720	6.2	5.11	16	35	4.982271233			174.4
96	Flora, Probat	Hansen Flora Danica/Danisco Probat	M	direct	commercial	morning	31.5	5	31.5	25	31.5	10	yes	8	47	yes	38	40	no	-		yes	-	720	5.65	5.2	16	38	4.741939078			180.2
97	Flora, Probat	Hansen Flora Danica/Danisco Probat	M	direct	commercial	morning	32.4	10	32.4	45	32.4	10	yes	12	32	yes	35	50	no	-		yes	-	720	6.4	5.11	16	35	4.969881644			173.9

98	MA400	Danisco 4001	MT	direct	commercial	morning	33	20	33	30	33	10	yes	12	33	yes	39.5	30	yes	39.5	25	yes	-	15	5.8		12	39.5	4.707570176	185.9
103	Alp Dip	Danisco 4001	MT	direct	commercial	morning	32	30	32	35	32	7	yes	10	32	yes	39.5	25	yes	39.5	12	-	-		6		12	39.5	4.696705781	185.5
133	2 Direct starters, TA19 and TAC (thermophilic; each preactivated for 30 mins at 31 °C)	TA19 (unknown), TAC (Lyo Pro)	T	direct	commercial	morning	31	40	31.5	32.5	32	20	Yes	10	42.5	No	38.5	30	Yes	38.5	30	yes	34	960	5.7		5,5 to 6	38.5	4.937267072	190.1
1	No				no	-	30	40	37	32	34.6	24	No	-	-	Yes	34.6	22	Yes	36.5	17	yes		420		5.30	14	37	4.522444234	167.3
2	No				no	-	30	42	34.5	55	33.5	8	No	-	-	Yes	46.5	32	No			yes	43	720			14	46.5	4.711132072	219.1
4	No				no	-	30	40	37	32	34.6	24	No	-	-	Yes	34.6	22	Yes	36.5	17	yes				5.40	14	37	3.908485019	144.6
6	No				no	-		40	36	22	36	7	No	-	-	Yes	36	18	Yes	42	36	yes	41	720			14	42	4.703978825	197.6
9	Natural milk culture				natural	morning	38	30	38	15	38	5	No	-	-	Yes	40	5	Yes	40	5	yes	35	420	6.00	5.30	4	40	4.857332496	194.3
12	Natural milk culture				natural	morning	37	20	39	55	38.8		No	-	-	Yes	42.6	10	Yes	42.6	10	yes	30	240	5.42	5.17	4	42.6	4.689308859	199.8
15	Natural milk culture				natural	morning	37	5	37	43	38	20	No	-	-	Yes	38	16	Yes	1	25	yes	39	260	5.21	4.95	4	39	4.345177617	169.5
17	No				no	morning	38	40	38	10	38	25	No	-	-	Yes	43	5	Yes	40	20	yes	30	100	6.10	5.30	4	43	4.079181246	175.4
19	Natural milk culture				natural	morning	40	30	38	15	37	5	No	-	-	Yes	40	5	Yes	40	6	yes	33	410	5.80	5.30	4	40	4.854063012	194.2
21	No				no	-	35		35	30	27		No	-	-	Yes	38/41		Yes	38	5	yes		180				39.5	4.11058971	162.4
24	No				no	-			35	50			No	-	-							no				5.20		35	3.477121255	121.7
63	-					-	-	-	-	-	-	-	-	-	-	-	-	-	-	-		-	-		-		-			0.0
99	-					-	-	-	-	-	-	-	-	-	-	-	-	-	-	-	-	-	-	-	-		-			0.0
100	-					-	-	-	-	-	-	-	-	-	-	-	-	-	-	-	-	-	-	-	-		-			0.0
101	-					-	-	-	-	-	-	-	-	-	-	-	-	-	-	-	-	-	-	-	-		-			0.0
102	-					-	-	-	-	-	-	-	-	-	-	-	-	-	-	-	-	-	-	-	-		-			0.0

Notes:

Table elements were left blank if no data were available or it made no sense to fill them in. [2)] Producers considered the storage of the evening milk as pre-maturing; [3)] values calculated to draw Figure 3.

Table S2c: Samples: coagulase-positive staphylococci (CPS) and staphylococcal enterotoxin (SE) data (sorted by "culture: direct or bulk," Table 2b, and "No."). Samples 13 and 135 (bold) were SE-positive. Samples 31, 90, and 92 (bold italic) were excluded from the statistical evaluation because of their high heat load. *S. aureus* genotype B (GTB).

General Information	Analysis				
Sample ID	CPS	CPS0	log(CPS0)	CPS >10^3	Enterotoxin SEA-SEE genotypes of the strains MRSA MLST
Sample No	[CFU/g]	[CFU/g]	log(CPS0)	Yes/No	Yes/No GTB (adlb pos) Strain 1 Strain 2 Strain 3 Strain 4 Strain 5 Yes/No Strain 1 Strain 2 Strain 3
34	<10	0	0	no	
38	<10	0	0	no	
39	<10	0	0	no	
40	<10	0	0	no	
41	280	280	2.44870632	no	
42	270	270	2.432969291	no	
43	<10	0	0	no	
44	<10	0	0	no	
50	<10	0	0	no	
51	<10	0	0	no	
52	<10	0	0	no	
56	28'000	28'000	4.447173542	Yes	NoNo P, C_I
62	130'000	130'000	5.113946693	Yes	NoNo F_I, F
72	17'000	17'000	4.230474467	Yes	NoNo R, A, A_II
73	29'000	29'000	4.462412973	Yes	NoYes B (adlb pos), M, F
74	54'000	54'000	4.732401802	Yes	NoNo J_I, AA, BS_I
75	17'000	17'000	4.230474467	Yes	NoNo J_I, AA, M
93	1'700	1'700	3.230704314	Yes	NoNo R
104	<10	0	0	No	No
105	<10	0	0	No	No
106	<10	0	0	No	No

107	<10	0	0	No	No
108	<10	0	0	No	No
109	<40	0	0	No	No
110	<10	0	0	No	No
111	<10	0	0	No	No
112	<10	0	0	No	No
113	<10	0	0	No	No
114	<10	0	0	No	No
115	<10	0	0	No	No
116	<10	0	0	No	No
117	<10	0	0	No	No
118	<10	0	0	No	No
119	<10	0	0	No	No
120	<10	0	0	No	No
121	<10	0	0	No	No
122	<10	0	0	No	No
124	<100	0	0	No	No
125	<400	0	0	No	No
127	<100	0	0	No	No
128	<100	0	0	No	No
131	1700	1700	3.230704314	Yes	NoYes B, R, RX
132	210000	210000	5.322221363	Yes	NoNo F, AO_III
**135**	**270000**	**270000**	**5.431365373**	**Yes**	**YesYes B, B_I**
35	120	120	2.08278537	no	
80	120'000	120'000	5.079184865	Yes	NoNo B (adlb neg), C, R, AF_I
123	2600	2600	3.415140352	Yes	NoYes B
126	600	600	2.778874472	No	No
129	< 400	0	0	No	No

130	< 100	0	0	No	No
134	20000	20000	4.30105171	Yes	NoNo RX, A_I
3	7100	7100	3.851319513	Yes	NoYes M1 (GTB_III) M2 (GTB_III) M4 (GTB_III) M5 (GTB_III)
5	<100	0	0	No	
7	<100	0	0	No	
8	230000	230000	5.361729724	Yes	NoYes SP1 (GTB_III) SP2 (GTB_III) SP3 (GTB_III) SP4 (GTB_III)
10	<100	0	0	No	
11	>300.000	3.00E+05	5.477122702	Yes	No BE5 (GTR)
**13**	**>300.000**	**3.00E+05**	**5.477122702**	**Yes**	**Yes Yes BL4 (GTB) BL9 (GTB)**
14	210000	210000	5.322221363	Yes	NoYes SA1 (GTR) SA2 (GTB) SA3 (GTB) SA4 (GTB) SA5 (GTB)
16	17000	17000	4.230474467	Yes	NoYes SA6 (GTB_I) SA7 (GTB) SA8 (GTB) SA9 (GTB) SA10 (GTB)
18	250000	250000	5.397941746	Yes	NoNo RO6 (GTI_I) RO7 (GTI) RO8 (GTI_I) RO9 (GTI_I) RO10 (GTI)
20	<100	0	0	No	
22	<100	0	0	No	
23	303000	303000	5.481444062	Yes	NoNo BK2 (GTA_I) BK3 (GTA_I) BK5 (GTA_I)
25	604000	604000	5.781037658	Yes	NoNo BK6 (GTA_I)
26	>1.000.000	1.00E+06	6.000000434	Yes	NoNo BK9 (GTA_I) BK10 (GTA_I) BK11 (GTA_I) BK13 (GTA_I)
27	>1.000.000	1.00E+06	6.000000434	Yes	NoNo BK14 (GTA_I) BK15 (GTA_I) BK16 (GTA_I) BK18 (GTA_I)
28	624000	624000	5.795185286	Yes	NoNo BK19 (GTA_I) BK21 (GTA_I) BK22 (GTA_I) BK23 (GTA_I)
29	<100	0	0	No	
30	<100	0	0	No	
31	***<10***	***0***	***0***	***no***	
32	<10	0	0	no	
33	<10	0	0	no	
36	<10	0	0	no	
37	5'600	5'600	3.748265573	Yes	NoNo Z, AK
45	49'000	49'000	4.690204943	Yes	NoNo R_I, R, I_I
46	<10	0	0	no	

47	<10	0	0	no	
48	<10	0	0	no	
49	300	300	2.478566496	no	
53	<10	0	0	no	
54	1'400	1'400	3.146438135	Yes	NoNo R
55	<10	0	0	no	
57	11'000	11'000	4.041432165	Yes	NoNo A_II
58	<10	0	0	no	
59	24'000	24'000	4.380229337	Yes	NoNo S, O
60	5'400	5'400	3.732474177	Yes	NoNo BS
61	290	290	2.463892989	no	
71	<10	0	0	no	
76	<10	0	0	no	
77	620	620	2.7930916	no	
78	160	160	2.206825876	no	
79	62'000	62'000	4.792398694	Yes	NoNo J
81	19'000	19'000	4.278776458	Yes	NoYes B, B_III
82	<10	0	0	no	
83	<10	0	0	no	
84	<10	0	0	no	
85	<10	0	0	no	
86	310	310	2.492760389	no	
87	260'000	260'000	5.414975018	Yes	NoYes R, B
88	<10	0	0	no	
89	39'000	39'000	4.591075743	Yes	NoYes B, BE
90	***<10***	***0***	***0***	***no***	
92	***<10***	***0***	***0***	***no***	
95	<10	0	0	no	

96	200	200	2.303196057	no	
97	<10	0	0	no	
98	1’500	1’500	3.176380692	Yes	NoNo AO
103	330’000	330’000	5.518515256	Yes	NoNo R
133	<100	0	0	No	No
1	148000	148000	5.17026465	Yes	NoYes O1 (GTB_III) O2 (GTB_III) O5 (GTB_III)
2	47000	47000	4.672107098	Yes	NoYes G1 (GTB_III) G2 (GTB_III) G3 (GTB_III) G4 (GTB_III) G5 (GTB_III)
4	167000	167000	5.222719072	Yes	NoYes O2_1 (GTR) O2_2 (GTB_III) O2_3 (GTB_III) O2_4 (GTB_III) O2_5 (GTB_III)
6	<100	0	0	No	
9	<100	0	0	No	
12	>300.000	3.00E+05	5.477122702	Yes	NoYes BL1 (GTB_III) BL7 (GTB)
15	530000	530000	5.724276689	Yes	NoNo BE6 (GTI_I) BE7 (GTI_I) BE8 (GTI_I) BE9 (GTI_I) BE10 (GTI_I)
17	82000	82000	4.913819149	Yes	NoNo RO1 (GTI_I) RO2 (GTI_I) RO3 (GTI) RO4 (GTI_I) RO5 (GTI_I)
19	<100	0	0	No	
21	4400	4400	3.643551369	Yes	NoNo B1 (GTAY) B2 (GTAY) B3 (GTAY) B4 (GTAY)
24	26000	26000	4.414990051	Yes	NoYes GB1 (GTB) GB2 (GTB) GB3 (GTR) GB4 (GTR) GB5 (GTB)
63	<10	0	0	no	
99	4’700	4’700	3.672190251	Yes	NoNo J_I, AA
100	450	450	2.654176542	no	
101	50’000	50’000	4.69897869	Yes	NoNo J_I, AA
102	23’000	23’000	4.361746718	Yes	NoNo F, F_I

Notes:

Table elements were left blank if no data were available or it made no sense to fill them in.

Table S3: Characteristic median and mean data: flora values (FVs) and hurdle indices (HIs) of the cheeses from the three countries and the eight regions (see also Figure 1, Austria (AT), Italy (IT), and Switzerland (CH)). Coagulase-positive staphylococci (CPS), staphylococcal enterotoxin (SE).

			AT			CH				IT			
				AT1	AT2		CH1	CH2	CH3		IT1	IT2	IT3
	n [-]		32	13	19	61	13	26	22	30	7	13	10
	log CPS [log CFU/g]	Range	0.5.431	0…5.431	0…0	0…5.519	0…5.519	0…5.415	0…5.114	0…6.000	0…5.223	0…5.724	0…6.000
	log CPS [log CFU/g]	MED	0	0	0	2.303	0	0	3.887	4.793	3.851	5.322	4.948
	CPS [CFU/g]	Range	<10…270'000	<10…270'000	<10…<10	<10…330'000	<10…330'000	<10…260'000	<10…130'000	<100…>1'000'000	<100…167'000	<100…530'000	<100…1'000'000
	CPS [CFU/g]	MED	11'300	11'300	<10	8'300	850	1'700	17'000	167'000	57'500	220'000	303'000
	SE [-]		yes	yes	no	no	No	No	No	yes	no	Yes	No
hurdle 1: temp	Temp (max) [°C]	Range	33…52	33…39	52…53	28…62	35…60	41…67	28…62	35…46.5	37…46.5	39…43.6	35…43.5
	Temp (max) [°C]	MED	52.0	37.0	52.0	45.0	42.0	45.0	40.5	41.8	42.0	41.0	42.5
hurdle 2: a(w)	Ripening [weeks]	Range	4…78	4…6.5	12…78	1…24	3…16	3…24	1…8	4…24	14…14	4…4	4…24
	Ripening [weeks]	MED	12.0	5.50	24.00	7.00	9.00	6.00	4.00	4.00	14.00	4.00	24.00
hurdle 3: pH	pH (min) [-]	Range	5.3…6.5 (7)	5.3…6.5 (7)	(7…7)	4.4…6.3 (7)	5.11…6 (7)	5.1…6.3 (7)	4.2…5.4 (7)	4.94…5.84 (7)	5.3…5.4 (7)	4.95…5.84	5.2…5.67 (7)
	pH (min) [-]	MED	(7.00)	5.80	(7.00)	5.30	5.80	5.30	5.33	5.34	(7.00)	5.30	5.40
hurdle 4: competitive flora	Flora Value FV [-]	mean	1.0	1.0	1.0	1.0	1.0	1.0	1.0	0.50	0.1	1.0	0.1
Hurdle Index HI "Temp (max)* FV*ripening/pH"			45.40	34.72	178.29	38.65	79.00	48.84	6.31	19.34	9.77	30.71	19.29

(7) pH 7 was given as a worst-case value when no pH values were available.

Table S4: Conover–Iman test results for all pairwise comparisons of differences in log(CPS), coagulase-positive staphylococci (CPS), between cheese types HC (n=19), SHC (n=98), and FC (n=6). Significant differences were found between HC and FC and between HC and SHC. Fresh cheese (FC), hard cheese (HC), semi-hard cheese (SHC).

Group(i)	Group(j)	Statistic	p-Value
FC	HC	2.818	0.006
FC	SHC	0.467	0.641
HC	SHC	4.481	0.000

Table S5: Conover–Iman test results for all pairwise comparisons of differences in log(CPS), coagulase-positive staphylococci (CPS), between cheese varieties. AOP/POD (Product of Designated Origin).

Group (i)	Group (j)	Statistic	p-Value
Alpkäse halbhart	Bergkäse	2.569	0.012
Alpkäse halbhart	Büscion, Büscion di Capra	1.053	0.294
Alpkäse halbhart	Formagella	2.910	0.004
Alpkäse halbhart	Formaggio di Alpe Ticinese AOP	2.874	0.005
Alpkäse halbhart	Minadur	1.319	0.190
Alpkäse halbhart	Mutschli/Tomme du Valais	0.759	0.450
Alpkäse halbhart	Raclette d’alpage	1.627	0.107
Alpkäse halbhart	Schnittkäse	0.042	0.966
Alpkäse halbhart	Tilsiter	2.019	0.046
Alpkäse halbhart	Toma	3.122	0.002
Alpkäse halbhart	Vacherin Fribourgeois alpage AOP	0.608	0.544
Bergkäse	Büscion, Büscion di Capra	2.768	0.007
Bergkäse	Formagella	5.098	0.000
Bergkäse	Formaggio di Alpe Ticinese AOP	4.597	0.000
Bergkäse	Minadur	3.024	0.003
Bergkäse	Mutschli/Tomme du Valais	1.023	0.308
Bergkäse	Raclette d’alpage	2.998	0.003
Bergkäse	Schnittkäse	2.143	0.034
Bergkäse	Tilsiter	2.761	0.007
Bergkäse	Toma	4.958	0.000
Bergkäse	Vacherin Fribourgeois alpage AOP	0.651	0.516
Büscion, Büscion di Capra	Formagella	0.972	0.333
Büscion, Büscion di Capra	Formaggio di Alpe Ticinese AOP	1.379	0.171
Büscion, Büscion di Capra	Minadur	0.212	0.833

Group (i)	Group (j)	Statistic	p-Value
Büscion, Büscion di Capra	Mutschli/Tomme du Valais	1.444	0.152
Büscion, Büscion di Capra	Raclette d’alpage	0.679	0.499
Büscion, Büscion di Capra	Schnittkäse	0.912	0.364
Büscion, Büscion di Capra	Tilsiter	1.505	0.135
Büscion, Büscion di Capra	Toma	1.447	0.151
Büscion, Büscion di Capra	Vacherin Fribourgeois alpage AOP	1.187	0.238
Formagella	Formaggio di Alpe Ticinese AOP	0.673	0.502
Formagella	Minadur	0.716	0.475
Formagella	Mutschli/Tomme du Valais	2.717	0.008
Formagella	Raclette d’alpage	0.008	0.994
Formagella	Schnittkäse	2.342	0.021
Formagella	Tilsiter	1.149	0.253
Formagella	Toma	0.725	0.470
Formagella	Vacherin Fribourgeois alpage AOP	2.011	0.047
Formaggio di Alpe Ticinese AOP	Minadur	1.160	0.249
Formaggio di Alpe Ticinese AOP	Mutschli/Tomme du Valais	2.870	0.005
Formaggio di Alpe Ticinese AOP	Raclette d’alpage	0.470	0.639
Formaggio di Alpe Ticinese AOP	Schnittkäse	2.514	0.013
Formaggio di Alpe Ticinese AOP	Tilsiter	0.844	0.401
Formaggio di Alpe Ticinese AOP	Toma	0.001	0.999
Formaggio di Alpe Ticinese AOP	Vacherin Fribourgeois alpage AOP	2.264	0.026
Minadur	Mutschli/Tomme du Valais	1.655	0.101
Minadur	Raclette d’alpage	0.498	0.619
Minadur	Schnittkäse	1.150	0.253
Minadur	Tilsiter	1.399	0.165
Minadur	Toma	1.217	0.226

Group (i)	Group (j)	Statistic	p-Value
Minadur	Vacherin Fribourgeois alpage AOP	1.351	0.179
Mutschli/Tomme du Valais	Raclette d’alpage	1.910	0.059
Mutschli/Tomme du Valais	Schnittkäse	0.710	0.479
Mutschli/Tomme du Valais	Tilsiter	2.227	0.028
Mutschli/Tomme du Valais	Toma	3.013	0.003
Mutschli/Tomme du Valais	Vacherin Fribourgeois alpage AOP	0.069	0.945
Raclette d’alpage	Schnittkäse	1.488	0.140
Raclette d’alpage	Tilsiter	1.058	0.292
Raclette d’alpage	Toma	0.486	0.628
Raclette d’alpage	Vacherin Fribourgeois alpage AOP	1.630	0.106
Schnittkäse	Tilsiter	1.962	0.052
Schnittkäse	Toma	2.678	0.009
Schnittkäse	Vacherin Fribourgeois alpage AOP	0.597	0.551
Tilsiter	Toma	0.854	0.395
Tilsiter	Vacherin Fribourgeois alpage AOP	2.103	0.038
Toma	Vacherin Fribourgeois alpage AOP	2.328	0.022

Table S6: Characteristic data of clouds 1 to 5, with each cloud containing cheese varieties showing no significant differences in log(CPS), n=123. Coagulase-positive staphylococci (CPS), staphylococcal enterotoxin (SE).

			Cloud 1 "Bergkäse"	Cloud 2 "Mutschli/Tomme"	Cloud 3 "Schnittkäse"	Cloud 4 "Tilsiter"	Cloud 5 "Formagella"
	n [-]		29	62	59	31	56
	log CPS [log CFU/g]	Range	0…3.7483	0…5.4150	0…5.5185	0…5.5185	0…>6
	log CPS [log CFU/g]	MED	0	0	0	2.7789	4.2305
	CPS [CFU/g]	Range	<10…5’600	<10…260’000	<10…330’000	<10…330’000	<10…>1’000’000
	CPS [CFU/g]	MED	<10	<10	<10	600	17’000
	SE [-]		no	no	no	yes, 1x [Tilsiter, 270’000 CPS, temp (max) 37 °C, pH(min) 6.52]	yes, 2x [Tilsiter, 270’000 CPS, temp (max) 37 °C, pH(min) 6.52; Formagella, >300’000 CPS, temp (max) 43.6 °C, pH(min) 5.84]
hurdle 1: temp	Temp (max) [°C]	Range	35…65	23.8.67	23.8…67	23.8…62	23.8…62
	Temp (max) [°C]	MED	52.00	44.00	42.00	38.00	42.00
hurdle 2: a(w)	Ripening [weeks]	Range	3…78	1…24	1…24	1…14	1…24
	Ripening [weeks]	MED	12.0	6.0	8.0	6.3	4.0
hurdle 3: pH	pH (min) [-]	Range	5.0…5.4 (7)	4.2…6.3 (7)	4.2…6.3 (7)	4.2…6.52	4.2…6.52 (7)
	pH (min) [-]	MED	7.00	5.40	5.36	5.80	5.38
hurdle 4: competitive flora	Flora value FV [-]	mean	0.917	0.890	0.885	0.797	0.675
Hurdle Index HI "Temp (max)*FV*ripening/pH"			81.74	43.51	55.48	32.90	21.1

Table S7: Conover–Iman test results for all pairwise comparisons of differences in log(CPS), coagulase-positive staphylococci (CPS), between the temperature tolerance of the cultures [n=94, no significant differences; mesophilic (M), n=12; thermophilic (T), n=20; and meso-thermophilic (MT), n=62], culture starter [n=107, significant difference between bulk and direct; bulk, n=44; bulk + direct, n=7; direct, n=56], and culture origin [n=112, significant difference between commercial and no; "-," n=2; commercial, n=95; natural, n=8; and no, n=7).

Temperature tolerance of the cultures			
Group(i)	Group(j)	Statistic	p-Value
M	MT	0.293	0.770
M	T	0.276	0.783
MT	T	0.751	0.454

Culture starter			
Group(i)	Group(j)	Statistic	p-Value
bulk	bulk + direct	1.667	0.098
bulk	direct	2.837	0.005
bulk + direct	direct	0.267	0.790

Culture origin			
Group(i)	Group(j)	Statistic	p-Value
-	commercial	1.140	0.257
-	natural	1.052	0.295
-	no	2.273	0.025
commercial	natural	0.047	0.963
commercial	no	2.573	0.011
natural	no	1.914	0.058

Table S8: Conover–Iman test results for all pairwise comparisons of differences in "pH before salting" between the temperature tolerance of the cultures [n=38, significant differences between M and MT, and between M and T; mesophilic (M), n=5; thermophilic (T), n=5; and meso-thermophilic (MT), n=28], culture starter [n=59, no significant differences; bulk, n=10; bulk + direct, n=2; direct, n=39, .","" n=8], and culture origin [n=59, no significant difference; commercial, n=45; natural n=4; no, n=4; and .","" n=6].

Temperature tolerance of the cultures			
**Group(i)**	**Group(j)**	**Statistic**	**p-Value**
M	MT	2.363	0.024
M	T	3.110	0.004
MT	T	1.689	0.100

Culture starter			
**Group(i)**	**Group(j)**	**Statistic**	**p-Value**
bulk	bulk + direct	0.293	0.771
bulk	direct	0.198	0.844
bulk		.	.
bulk + direct	direct	0.216	0.830
bulk + direct		.	.
direct		.	.

Culture origin			
**Group(i)**	**Group(j)**	**Statistic**	**p-Value**
commercial	natural	0.238	0.813
commercial	no	1.410	0.165
commercial		.	.

Culture origin			
Group(i)	Group(j)	Statistic	p-Value
natural	no	1.216	0.230
natural		.	.
no		.	.

Table S9: Flora values (FVs) and hurdle indices (His) of all cheeses, sorted by HI. Samples 13 and 135 (bold) were staphylococcal enterotoxin (SE)-positive. Samples 31, 90, and 92 (bold italic) were excluded from the statistical evaluation because of their high heat load. Austria (AT), Italy (IT), and Switzerland (CH), AOP/POD (Product of Designated Origin).

General information						Hurdle Index				
Sample No.	Cheese Type	Name of the product	Country							
No.	FC, SHC, HC	Variety name	AT, CH, IT	Region name	Region group	Flora value (FV)	Temp (max) [°C]	Ripening [weeks]	pH	Hurdle index (HI) "temp (max)*FV*rip-
58	FC	Büscion, Büscion di Capra and Tomino di Capra	CH	Ticino	CH3	1	61.00		4.50	0.00
92	***FC***	***Büscion, Büscion di Capra and Tomino di Capra***	***CH***	***Ticino***	***CH3***	***1***	***65.00***		***7***	***0.00***
63	SHC	Formaggio d'alpe ticinese AOP	CH	Ticino	CH3	0.1	0.00		7	0.00
120	HC	Bergkäse	AT	Vorarlberg	A2	0.1	52.30		7	0.00
100	FC	Büscion, Büscion di Capra and Tomino di Capra	CH	Ticino	CH3	0.1	0.00		7	0.00
126	SHC	Schnittkäse	AT	Tirol	A1	1	33.00		5.8	0.00
93	SHC	Alpkäse halbhart	CH	Graubünden	CH2	1	42.50		7	0.00
21	SHC	Toma	IT	Verbania, Cusio, Ossola	I3	0.1	39.50	0	7	0.00
99	FC	Büscion, Büscion di Capra and Tomino di Capra	CH	Ticino	CH3	0.1	0.00		7	0.00
60	FC	Büscion, Büscion di Capra and Tomino di Capra	CH	Ticino	CH3	1	23.80		4.40	0.00
57	SHC	Formagella	CH	Ticino	CH3	1	38.00		7	0.00
75	SHC	Formaggio d'alpe ticinese AOP	CH	Ticino	CH3	1	45.00		5.30	0.00
102	SHC	Formaggio d'alpe ticinese AOP	CH	Ticino	CH3	0.1	0.00		7	0.00
24	SHC	Toma	IT	Verbania, Cusio, Ossola	I3	0.1	35.00	0	5.20	0.00
101	SHC	Formagella	CH	Ticino	CH3	0.1	0.00		7	0.00
62	SHC	Formaggio d'alpe ticinese AOP	CH	Ticino	CH3	1	46.00		7	0.00

127	SHC	Schnittkäse	AT	Tirol	A1	0.1	37.00	4	5.65	2.62
17	SHC	Formagella	IT	Valcamonica - Brescia	I2	0.1	43.00	4	5.30	3.25
79	FC	Büscion, Büscion di Capra and Tomino di Capra	CH	Ticino	CH3	1	28.00	1	4.62	6.06
78	FC	Büscion, Büscion di Capra and Tomino di Capra	CH	Ticino	CH3	1	28.00	1	4.27	6.56
85	SHC	Alpkäse halbhart	CH	Graubünden	CH2	1	8.00	6	7	6.86
6	SHC	Minadur	IT	Val Brembana - Bergamo	I1	0.1	42.00	14	7	8.40
2	SHC	Minadur	IT	Val Brembana - Bergamo	I1	0.1	46.50	14	7	9.30
4	SHC	Minadur	IT	Val Brembana - Bergamo	I1	0.1	37.00	14	5.40	9.59
1	SHC	Minadur	IT	Val Brembana - Bergamo	I1	0.1	37.00	14	5.30	9.77
32	SHC	Mutschli/Tomme du Valais	CH	Bern	CH1	1	35.00	3	7	15.00
61	SHC	Formagella	CH	Ticino	CH3	1	35.00	3	7	15.00
9	SHC	Formagella	IT	Valcamonica - Brescia	I2	0.5	40.00	4	5.30	15.09
19	SHC	Formagella	IT	Valcamonica - Brescia	I2	0.5	40.00	4	5.30	15.09
15	SHC	Formagella	IT	Valcamonica - Brescia	I2	0.5	39.00	4	4.95	15.76
12	SHC	Formagella	IT	Valcamonica - Brescia	I2	0.5	42.60	4	5.17	16.48
26	SHC	Toma	IT	Verbania, Cusio, Ossola	I3	0.1	41.60	24	5.55	17.99
27	SHC	Toma	IT	Verbania, Cusio, Ossola	I3	0.1	43.50	24	5.67	18.41
28	SHC	Toma	IT	Verbania, Cusio, Ossola	I3	0.1	42.50	24	5.30	19.25
23	SHC	Toma	IT	Verbania, Cusio, Ossola	I3	0.1	43.50	24	5.4	19.33
25	SHC	Toma	IT	Verbania, Cusio, Ossola	I3	0.1	42.50	24	5.27	19.35
30	SHC	Toma	IT	Verbania, Cusio, Ossola	I3	0.1	43.50	24	5.30	19.70
**135**	**SHC**	**Tilsiter**	**AT**	**Tirol**	**A1**	**1**	**37.00**	**4**	**6.52**	**22.70**
71	SHC	Mutschli/Tomme du Valais	CH	Bern	CH1	1	55.00	3	7	23.57
22	SHC	Toma	IT	Verbania, Cusio, Ossola	I3	1	42.00	4	7	24.00
36	SHC	Mutschli/Tomme du Valais	CH	Bern	CH1	1	60.00	3	7	25.71
76	SHC	Mutschli/Tomme du Valais	CH	Bern	CH1	1	60.00	3	7	25.71
53	SHC	Mutschli/Tomme du Valais	CH	Graubünden	CH2	1	45.00	4	7	25.71
77	SHC	Formagella	CH	Ticino	CH3	1	35.00	4	5.20	26.92

37	SHC	Mutschli/Tomme du Valais	CH	Uri	CH2	1	44.00	3.5	5.40	28.52
84	SHC	Büscion, Büscion di Capra and Tomino di Capra	CH	Ticino	CH3	1	62.00	2	4.20	29.52
**13**	**SHC**	**Formagella**	**IT**	**Valcamonica - Brescia**	**I2**	**1**	**43.60**	**4**	**5.84**	**29.86**
14	SHC	Formagella	IT	Valcamonica - Brescia	I2	1	41.00	4	5.34	30.71
11	SHC	Formagella	IT	Valcamonica - Brescia	I2	1	41.00	4	5.34	30.71
8	SHC	Formagella	IT	Valcamonica - Brescia	I2	1	41.50	4	5.39	30.80
31	***SHC***	***Mutschli/Tomme du Valais***	***CH***	***Bern***	***CH1***	***1***	***72.00***	***3***	***7***	***30.86***
16	SHC	Formagella	IT	Valcamonica - Brescia	I2	1	41.00	4	5.29	31.00
20	SHC	Formagella	IT	Valcamonica - Brescia	I2	1	40.00	4	5.10	31.37
10	SHC	Formagella	IT	Valcamonica - Brescia	I2	1	40.00	4	5.10	31.37
134	SHC	Schnittkäse	AT	Tirol	A1	1	37.00	5	5.8	31.90
125	SHC	Schnittkäse	AT	Tirol	A1	1	38.00	5	5.9	32.20
29	SHC	Toma	IT	Verbania, Cusio, Ossola	I3	1	43.50	4	5.4	32.22
18	SHC	Formagella	IT	Valcamonica - Brescia	I2	1	43.30	4	5.30	32.68
42	SHC	Alpkäse halbhart	CH	Schwyz	CH2	0.5	46.00	10	7	32.86
132	SHC	Schnittkäse	AT	Kärnten	A1	1	39.00	5	5.9	33.05
83	SHC	Formagella	CH	Ticino	CH3	1	62.00	3	5.40	34.44
129	SHC	Schnittkäse	AT	Tirol	A1	1	38.00	5.5	6.02	34.72
55	SHC	Formagella	CH	Ticino	CH3	1	61.00	4	7	34.86
54	SHC	Alpkäse halbhart	CH	Graubünden	CH2	1	41.00	6	7	35.14
47	SHC	Alpkäse halbhart	CH	Graubünden	CH2	1	65.00	3	5.20	37.50
49	SHC	Mutschli/Tomme du Valais	CH	Graubünden	CH2	1	65.00	3	5.20	37.50
46	SHC	Alpkäse halbhart	CH	Graubünden	CH2	1	65.00	3	5.17	37.72
128	SHC	Schnittkäse	AT	Tirol	A1	1	36.00	6	5.7	37.89
131	SHC	Schnittkäse	AT	Tirol	A1	1	37.00	5.5	5.3	38.40
48	SHC	Alpkäse halbhart	CH	Graubünden	CH2	1	67.00	3	5.20	38.65
133	SHC	Schnittkäse	AT	Tirol	A1	1	38.50	5.75	5.7	38.84
130	SHC	Schnittkäse	AT	Tirol	A1	1	36.00	6.5	5.95	39.33

124	SHC	Schnittkäse	AT	Tirol	A1	1	38.00	5.5	5.3	39.43
88	SHC	Alpkäse halbhart	CH	Graubünden	CH2	1	44.00	5	5.30	41.51
89	SHC	Alpkäse halbhart	CH	Graubünden	CH2	1	44.00	5	5.30	41.51
119	HC	Bergkäse	AT	Vorarlberg	A2	0.5	52.00	12	7	44.57
121	HC	Bergkäse	AT	Vorarlberg	A2	0.5	52.00	12	7	44.57
123	SHC	Schnittkäse	AT	Tirol	A1	1	37.00	6.5	5.3	45.38
115	HC	Bergkäse	AT	Vorarlberg	A2	0.5	53.00	12	7	45.43
40	SHC	Alpkäse halbhart	CH	Graubünden	CH2	1	42.00	6	5.20	48.46
82	SHC	Raclette d’alpage	CH	Valais	CH1	1	43.00	8	7	49.14
33	SHC	Alpkäse halbhart	CH	Uri	CH2	1	42.00	6	5.12	49.22
45	SHC	Alpkäse halbhart	CH	Graubünden	CH2	1	45.00	6	5.30	50.94
50	SHC	Alpkäse halbhart	CH	Glarus	CH2	1	45.00	8	7	51.43
51	SHC	Alpkäse halbhart	CH	Glarus	CH2	1	45.00	8	7	51.43
52	SHC	Alpkäse halbhart	CH	Glarus	CH2	1	45.00	8	7	51.43
39	SHC	Alpkäse halbhart	CH	Glarus	CH2	1	45.50	8	7	52.00
59	SHC	Alpkäse halbhart	CH	Graubünden	CH2	1	46.00	6	5.10	54.12
43	SHC	Alpkäse halbhart	CH	Glarus	CH2	1	45.10	8	6.3	57.27
81	SHC	Raclette d’alpage	CH	Valais	CH1	1	42.00	8	5.36	62.69
74	SHC	Alpkäse halbhart	CH	Ticino	CH3	1	43.00	8	5.30	64.91
72	SHC	Formaggio d’alpe ticinese AOP	CH	Ticino	CH3	1	46.00	8	5.35	68.79
44	SHC	Alpkäse halbhart	CH	Glarus	CH2	1	44.80	8	5.20	68.92
73	SHC	Formaggio d’alpe ticinese AOP	CH	Ticino	CH3	1	46.00	8	5.30	69.43
56	SHC	Formaggio d’alpe ticinese AOP	CH	Ticino	CH3	1	45.00	8	5.17	69.63
38	SHC	Alpkäse halbhart	CH	Glarus	CH2	1	45.50	8	5.17	70.41
**90**	**SHC**	**Alpkäse halbhart**	**CH**	**Ticino**	**CH3**	**1**	**65.00**	**6**	**5.20**	**75.00**
103	SHC	Raclette d’alpage	CH	Valais	CH1	1	39.50	12	6	79.00
5	SHC	Minadur	IT	Val Brembana - Bergamo	I1	1	40.00	14	7	80.00
98	SHC	Raclette d’alpage	CH	Valais	CH1	1	39.50	12	5.8	81.72

35	SHC	Alpkäse halbhart	CH	Obwalden	CH1	1	47.30	9	5.20	81.87
80	SHC	Formaggio d'alpe ticinese AOP	CH	Ticino	CH3	1	55.00	8	5.30	83.02
41	SHC	Alpkäse halbhart	CH	Schwyz	CH2	1	45.00	10	5.18	86.87
3	SHC	Minadur	IT	Val Brembana - Bergamo	I1	1	44.00	14	7	88.00
122	HC	Bergkäse	AT	Vorarlberg	A2	1	52.00	12	7	89.14
117	HC	Bergkäse	AT	Vorarlberg	A2	1	52.00	12	7	89.14
114	HC	Bergkäse	AT	Vorarlberg	A2	1	52.00	12	7	89.14
118	HC	Bergkäse	AT	Vorarlberg	A2	1	52.00	12	7	89.14
116	HC	Bergkäse	AT	Vorarlberg	A2	1	53.00	12	7	90.86
7	SHC	Minadur	IT	Val Brembana - Bergamo	I1	1	46.00	14	7	92.00
97	SHC	Vacherin Fribourgeois alpage AOP	CH	Fribourg	CH1	1	35.00	16	5.11	109.59
95	SHC	Vacherin Fribourgeois alpage AOP	CH	Fribourg	CH1	1	35.00	16	5.11	109.59
34	SHC	Alpkäse halbhart	CH	Nidwalden	CH1	1	42.00	14	5.15	114.17
96	SHC	Vacherin Fribourgeois alpage AOP	CH	Fribourg	CH1	1	38.00	16	5.20	116.92
105	HC	Bergkäse	AT	Vorarlberg	A2	1	52.00	24	7	178.29
106	HC	Bergkäse	AT	Vorarlberg	A2	1	52.00	24	7	178.29
108	HC	Bergkäse	AT	Vorarlberg	A2	1	52.00	24	7	178.29
112	HC	Bergkäse	AT	Vorarlberg	A2	1	52.00	24	7	178.29
113	HC	Bergkäse	AT	Vorarlberg	A2	1	52.00	24	7	178.29
107	HC	Bergkäse	AT	Vorarlberg	A2	1	52.50	24	7	180.00
110	HC	Bergkäse	AT	Vorarlberg	A2	1	53.00	24	7	181.71
111	HC	Bergkäse	AT	Vorarlberg	A2	1	53.00	24	7	181.71
86	SHC	Alpkäse halbhart	CH	Graubünden	CH2	1	45.00	24	5.20	207.69
87	SHC	Alpkäse halbhart	CH	Graubünden	CH2	1	45.00	24	5.20	207.69
109	HC	Bergkäse	AT	Vorarlberg	A2	1	52.00	38	7	282.29
104	HC	Bergkäse	AT	Vorarlberg	A2	1	52.00	78	7	579.43

Table S10: Conover–Iman test results for all pairwise comparisons of differences in log(CPS), coagulase-positive staphylococci (CPS), between cheese varieties, SHC only, n=98. AOP/POD (Product of Designated Origin).

Group(i)	Group(j)	Statistic	p-Value
Alpkäse halbhart	Büscion di Capra	0.657	0.513
Alpkäse halbhart	Formagella	2.723	0.008
Alpkäse halbhart	Formaggio di Alpe Ticinese AOP	2.557	0.012
Alpkäse halbhart	Minadur	1.220	0.226
Alpkäse halbhart	Mutschli/Tomme du Valais	0.700	0.486
Alpkäse halbhart	Raclette d’alpage	1.483	0.142
Alpkäse halbhart	Schnittkäse	0.036	0.972
Alpkäse halbhart	Tilsiter	1.916	0.059
Alpkäse halbhart	Toma	3.036	0.003
Alpkäse halbhart	Vacherin Fribourgeois alpage AOP	0.584	0.561
Büscion, Büscion di Capra	Formagella	1.454	0.150
Büscion, Büscion di Capra	Formaggio di Alpe Ticinese AOP	1.606	0.112
Büscion, Büscion di Capra	Minadur	1.113	0.269
Büscion, Büscion di Capra	Mutschli/Tomme du Valais	0.348	0.729
Büscion, Büscion di Capra	Raclette d’alpage	1.311	0.193
Büscion, Büscion di Capra	Schnittkäse	0.655	0.514
Büscion, Büscion di Capra	Tilsiter	1.854	0.067
Büscion, Büscion di Capra	Toma	1.716	0.090
Büscion, Büscion di Capra	Vacherin Fribourgeois alpage AOP	0.272	0.787
Formagella	Formaggio di Alpe Ticinese AOP	0.503	0.616
Formagella	Minadur	0.683	0.496
Formagella	Mutschli/Tomme du Valais	2.533	0.013
Formagella	Raclette d’alpage	0.046	0.963

Group(i)	Group(j)	Statistic	p-Value
Formagella	Schnittkäse	2.195	0.031
Formagella	Tilsiter	1.102	0.274
Formagella	Toma	0.788	0.433
Formagella	Vacherin Fribourgeois alpage AOP	1.896	0.061
Formaggio di Alpe Ticinese AOP	Minadur	0.994	0.323
Formaggio di Alpe Ticinese AOP	Mutschli/Tomme du Valais	2.574	0.012
Formaggio di Alpe Ticinese AOP	Raclette d’alpage	0.388	0.699
Formaggio di Alpe Ticinese AOP	Schnittkäse	2.238	0.028
Formaggio di Alpe Ticinese AOP	Tilsiter	0.866	0.389
Formaggio di Alpe Ticinese AOP	Toma	0.201	0.841
Formaggio di Alpe Ticinese AOP	Vacherin Fribourgeois alpage AOP	2.053	0.043
Minadur	Mutschli/Tomme du Valais	1.530	0.130
Minadur	Raclette d’alpage	0.441	0.660
Minadur	Schnittkäse	1.067	0.289
Minadur	Tilsiter	1.340	0.184
Minadur	Toma	1.238	0.219
Minadur	Vacherin Fribourgeois alpage AOP	1.269	0.208
Mutschli/Tomme du Valais	Raclette d’alpage	1.746	0.084
Mutschli/Tomme du Valais	Schnittkäse	0.653	0.516
Mutschli/Tomme du Valais	Tilsiter	2.105	0.038
Mutschli/Tomme du Valais	Toma	2.897	0.005
Mutschli/Tomme du Valais	Vacherin Fribourgeois alpage AOP	0.084	0.933
Raclette d’alpage	Schnittkäse	1.358	0.178
Raclette d’alpage	Tilsiter	1.034	0.304
Raclette d’alpage	Toma	0.563	0.575
Raclette d’alpage	Vacherin Fribourgeois alpage AOP	1.509	0.135

Group(i)	Group(j)	Statistic	p-Value
Schnittkäse	Tilsiter	1.864	0.066
Schnittkäse	Toma	2.609	0.011
Schnittkäse	Vacherin Fribourgeois alpage AOP	0.571	0.569
Tilsiter	Toma	0.784	0.435
Tilsiter	Vacherin Fribourgeois alpage AOP	1.999	0.049
Toma	Vacherin Fribourgeois alpage AOP	2.257	0.027

Table S11: Characteristic data of clouds 2 to 6, with each cloud containing cheese varieties showing no significant differences in log(CPS), coagulase-positive staphylococci (CPS), if only semi-hard cheeses (SHC) were taken. pH (min): (7) was used as a worst case if no pH was mentioned. Staphylococcal enterotoxin (SE).

			Cloud 2, SHC only "Mutschli/Tomme"	Cloud 3, SHC only "Schnittkäse»"	Cloud 4, SHC only "Tilsiter"	Cloud 5, SHC only "Formagella"	Cloud 6, SHC only "Büscion"
	n [-]		56	53	49	39	24
	log CPS [log CFU/g]	Range	0…5.4150	0…5.5185	0…>6	0…5.7542	0…5.5185
	log CPS [log CFU/g]	MED	0	0	4.3617	4.2305	1.3894
	CPS [CFU/g]	Range	<10…260'000	<10…330'000	<10…>600'000	<10…530'000	<10…330'000
	CPS [CFU/g]	MED	<10	<10	23'000	17'000	300
	SE [-]		no	no	yes, 2x [Tilsiter, 270'000 CPS, temp (max) 37 °C, pH(min) 6.52; Formagella, >300'000 CPS, temp (max) 43.6 °C, pH(min) 5.84]	yes, 1x [Tilsiter, 270'000 CPS, temp (max) 37 °C, pH(min) 6.52]	no
hurdle 1: temp	Temp (max) [°C]	Range	33…67	33.62	35…62	35…62	33…62
	Temp (max) [°C]	MED	44.00	42.25	42.00	42.00	38.25
hurdle 2: a(w)	Ripening [weeks]	Range	2…24	2…24	3…24	2…14	2…14
	Ripening [weeks]	MED	6.0	8.0	8.0	4.0	6.5
hurdle 3: pH	pH (min) [-]	Range	4.2…6.3 (7)	4.2…6.3 (7)	4.95…5.84 (7)	4.2…6 (7)	4.2…6.0 (7)
	pH (min) [-]	MED	5.53	5.40	5.4	5.36	5.80
hurdle 4: competitive flora	Flora Value FV [-]	mean	0.911	0.906	0.665	0.764	0.813
	Hurdle Index "Temp (max)*FV*ripening/pH"		43.52	56.69	41.40	23.95	34.83
